# Major depression, physical health and molecular senescence markers abnormalities

**DOI:** 10.1038/s44220-023-00033-z

**Published:** 2023-03-22

**Authors:** Johanna Seitz-Holland, Benoit H. Mulsant, Charles F. Reynolds, Daniel M. Blumberger, Jordan F. Karp, Meryl A. Butters, Ana Paula Mendes-Silva, Erica L. Vieira, George Tseng, Eric J. Lenze, Breno S. Diniz

**Affiliations:** 1 Department of Psychiatry, Brigham and Women’s Hospital, Harvard Medical School, Boston, MA, USA.; 2 Department of Psychiatry, Massachusetts General Hospital, Harvard Medical School, Boston, MA, USA.; 3 Centre for Addiction and Mental Health, Toronto, Ontario, Canada.; 4 Department of Psychiatry, Temerty Faculty of Medicine, University of Toronto, Toronto, Ontario, Canada.; 5 Department of Psychiatry, University of Pittsburgh School of Medicine, Pittsburgh, PA, USA.; 6 Department of Psychiatry, The University of Arizona College of Medicine, Tucson, AZ, USA.; 7 Department of Biostatistics, University of Pittsburgh School of Public Health, Pittsburgh, PA, USA.; 8 Department of Psychiatry, Washington University School of Medicine, St Louis, MO, USA.; 9 UConn Center on Aging, University of Connecticut, Farmington, CT, USA.; 10 Department of Psychiatry, UConn School of Medicine, Farmington, CT, USA.

## Abstract

Previous studies suggested the role of cellular senescence in late-life depression (LLD). However, it is unclear how this finding relates to common features of LLD, such as medical and cognitive problems. We applied factor analyses to an extensive battery of clinical variables in 426 individuals with LLD. Here we tested the relationship between these factors, age and sex, with an index of cellular senescence based on 22 senescence-associated secretory phenotype proteins. We found four factors: ‘depression and anxiety severity’, ‘cognitive functioning’, ‘cardiovascular and cardiometabolic health’ and ‘blood pressure’. A higher senescence-associated secretory phenotype index was associated with poorer ‘cognitive functioning’ and ‘cardiovascular and cardiometabolic health’ but not with ‘depression and anxiety severity’. These findings highlight the role of cellular senescence in poorer physical and cognitive health in LLD. They are consonant with the viewpoint that co-occurring medical burdens and their associated disabilities are part of a phenotype of accelerated ageing in LLD.

Late-life depression (LLD) is a major depressive disorder in older adults. LLD is common and poses a substantial burden for affected individuals, their families and society^1^. These burdens include worse quality of life, impaired activities of daily living^2^ and increased frailty^3^. Previous studies demonstrated a strong link among LLD, poor physical health^4^, cognitive impairment, an increased risk for dementia and mortality^5^. These clinico-epidemiological findings suggest that older adults with LLD may experience an accelerated ageing phenotype^6^.

Accelerated ageing processes in LLD may be driven by increased allostatic load, altered proteostasis control, pro-inflammatory mechanisms and systemic oxidative stress^7,8^. Research also suggests that accelerated ageing in LLD may occur on the cellular and subcellular level^9^ and be linked to enhanced senescence processes^10^. Cellular senescence^11^ has emerged as a pivotal hallmark of the biology of ageing. It is a complex stress response in which cells irreversibly lose their proliferative capacity, become resistant to apoptosis^12^ and develop a multicomponent secretory phenotype^13^, referred to as the senescence-associated secretory phenotype (SASP)^12^. The SASP includes proteins involved in cycle control, intercellular communication, the immune-inflammatory response and tissue remodelling^14^. Under non-pathological conditions, SASP proteins are essential for embryonic development and tissue patterning throughout life^15^. However, the accumulation of senescent cells and the increased secretion of SASP proteins with age is linked to tissue deterioration and the emergence of physical disorders prevalent in older adults^16^. Human and animal studies found that the increased expression of SASP proteins drives multiple age-related phenotypes, such as atherosclerosis^17^, osteoarthritis^18^, cancer^19^, kidney dysfunction^20^ and a shortened health span^21^. In humans, an increase in SASP proteins is related to obesity, cardiometabolic dysregulation^22^ and frailty^14^. Several studies characterized the role of cellular senescence and SASP proteins in the brain. Aged microglia, astrocytes and neurons exhibit various features of senescence, including the expression of SASP proteins^23–25^. In Alzheimer’s disease, an upregulation of SASP proteins has been found in hippocampal neurons and astrocytes^26^.

These findings support that cellular senescence underlies bodywide accelerated ageing and that SASP proteins are markers for these processes. Since the SASP proteins reflect multiple interrelated biological functions, examining them as a biomarker-composite index (SASP index) might be more robust than investigating single proteins. While the SASP is cell dependent, there are common SASP proteins expressed by senescent cells. On the basis of these SASP markers^27^, our group developed a peripheral SASP index comprising 22 proteins. We validated the SASP index in several studies^28,29^ and consistently found a higher SASP index in individuals with LLD compared with healthy individuals^28,30,31^, suggesting that the SASP index might be a marker for accelerated ageing in LLD.

While these previous studies report an increase in the SASP index in LLD, it is unclear which clinical characteristics of LLD are linked to cellular senescence and the SASP index. We hypothesize that cognitive and physical health variables (for example, medical comorbidities and general physical health) are correlated with the increased SASP index and thus might drive accelerated ageing in LLD. Alternatively, psychopathology itself (for example, the severity of depression and psychiatric comorbidities) may drive the increased SASP. Testing this hypothesis may deepen our understanding of biological ageing abnormalities in LLD and help identify individuals who are particularly vulnerable to accelerated ageing and may benefit from targeted prevention and treatment strategies. In this Article, as a step towards this goal, we aimed to identify which clinical variables are related to the peripheral SASP index in LLD. We leveraged a relatively large LLD sample (*n* = 426) with SASP protein measurements. Following previous studies demonstrating the utility of integrating various variables^32–34^, we used factor analyses to group clinical variables. We then tested our hypothesis that the resulting clinical factors would be associated with the SASP index.

## Results

Participants were recruited as part of a clinical trial (the Incomplete Response in Late-Life Depression: Getting to Remission (IRL-GREY) study) conducted between August 2009 and August 2014, as detailed previously^35^ (Supplementary Table 1). We included 426 participants, aged 60 years or older, with a diagnosis of current non-psychotic major depressive disorder and a score ≥15 on the Montgomery–Asberg Depression Rating Scale (MADRS)^36^. All participants included in this analysis had complete demographic and protein data. Their mean age ± standard deviation (s.d.) was 68.94 ± 7.10 years, and 64% were female. For demographic and clinical information, see Tables 1 and 2, and for medication information, see Supplementary Table 2.

### Clinical variables

**Fig. 1 |:**
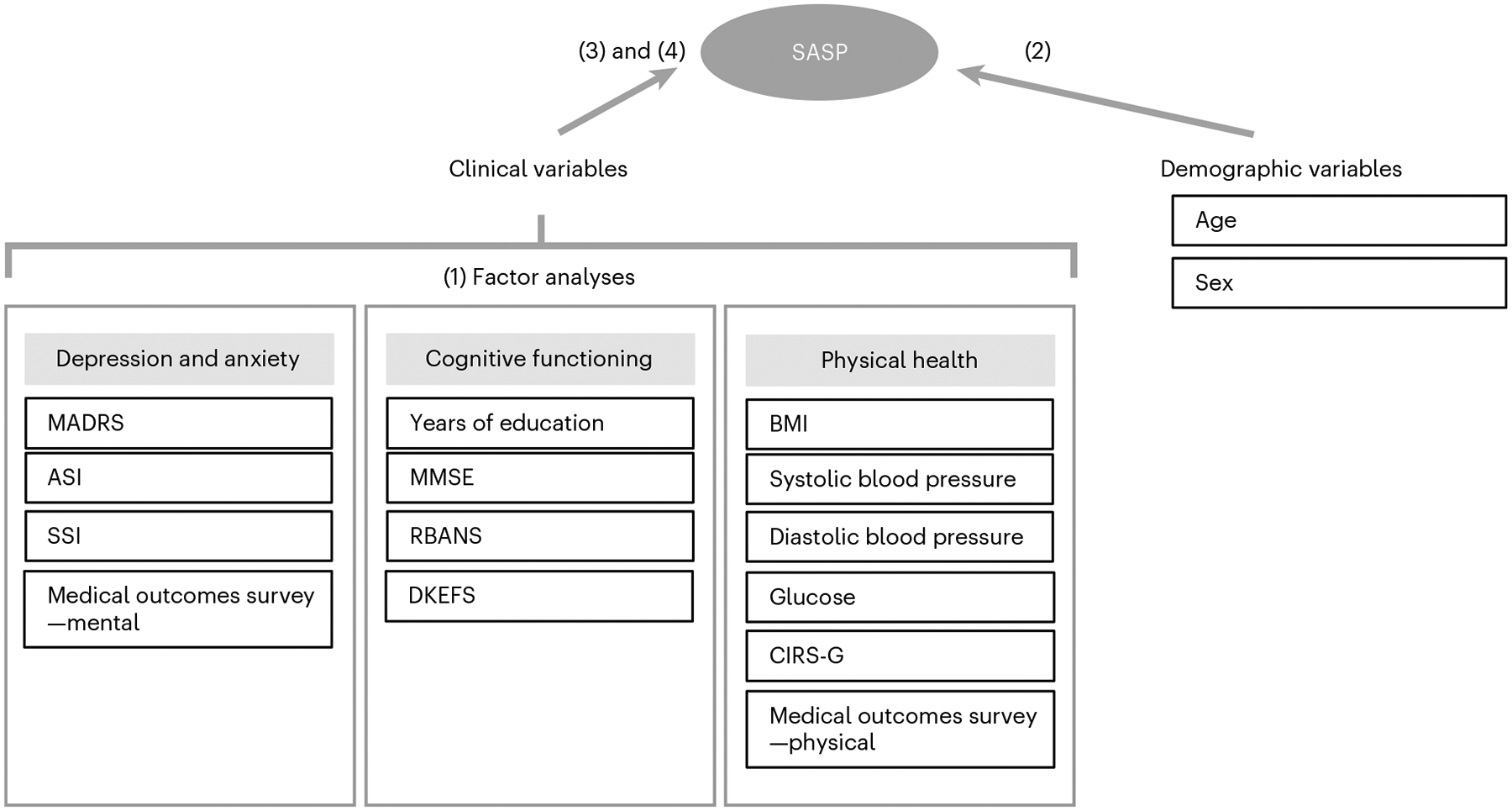
The statistical analysis strategy. First, we conducted three independent factor analyses to group clinical variables. Next, we calculated a regression to determine the effects of age and sex on the SASP index. We then utilized correlation and regression analyses to explore the association between the SASP index and the factors. Finally, we split the sample into participants with early-onset depression (age of onset before 65 years) or late-onset depression (age of onset at or after 65 years) and repeated the regression analyses for these two subgroups. References: MADRS^36^, ASI^37^, SSI^38^, Medical Outcomes Survey—mental^39^, MMSE^40^, RBANS^41^, DKEFS^42^, BMI, Medical Outcomes Survey—physical^39^ and SASP.

For an overview of our analytic strategy, see Fig. 1. We examined an extensive battery of clinical variables to characterize mental health, including MADRS^36^, Anxiety Sensitivity Index (ASI)^37^, Scale of Suicidal Ideation (SSI)^38^ and the Medical Outcomes Survey—mental^39^. To examine cognitive functioning, we recorded self-reported years of education, Mini-Mental Status Examination (MMSE)^40^, Repeatable Battery for the Assessment of Neuropsychological Status (RBANS)^41^ and the Delis–Kaplan Executive Function System (DKEFS)^42^. Last, we obtained anthropometric data, blood pressure, fasting glucose levels, Cumulative Illness Rating Scale—geriatrics (CIRS-G)^43^ and the Medical Outcomes Survey—physical^39^.

As presented in Supplementary Table 3, the factor analyses revealed one ‘depression and anxiety severity’ factor, one ‘cognitive functioning’ factor and two physical health-related factors. The first physical health-related factor is termed the ‘cardiovascular and cardiometabolic health’ factor. This explained the most variance and comprised body mass index (BMI), fasting glucose levels, CIRS-G and Medical Outcomes Survey—physical scales. The second physical health factor is termed ‘blood pressure’ because it comprises systolic and diastolic blood pressure.

### Relationship between the SASP and demographic variables

On the basis of previous pre-clinical studies focused on the changes in the secretome pattern of senescent cells^27^ and our previous publications^28,30^, we determined 22 SASP proteins and calculated a SASP index. We conducted a regression analysis with the SASP index as the dependent variable and age and sex as independent variables (*F*(2, 423) = 21.31, *P* < 0.001, adjusted *R*^2^ = 0.087 and power >0.99). This demonstrated a significant effect of age (standardized *β* = 0.24, *T* = 5.24, *P* < 0.001 and variance inflation factor of 1.00) and sex (standardized *β* = −0.19, *T* = −4.11, *P* < 0.001 and variance inflation factor of 1.00) so that older and male participants presented with a higher SASP index (Supplementary Fig. 1).

### Relationship between the SASP index and clinical variables

We used correlation and regression analyses to calculate the association between the SASP index and the factors determined above. A higher SASP index was correlated with worse cognitive functioning (*r* = −0.18, *P* < 0.001 and power 0.90) and worse cardiovascular and cardiometabolic health (*r* = 0.42, *P* < 0.001 and power >0.99) (Supplementary Table 4). Sex-specific correlations demonstrated a positive correlation between the SASP index and the cardiovascular and cardiometabolic health factor, both for males (*r* = 0.53, *P* < 0.001 and power >0.99) and females (*r* = 0.37, *P* < 0.001 and power >0.99), and a negative correlation between the SASP index and the cognitive functioning factor for females (*r* = −0.17, *P* = 0.005 and power 0.63).

As shown in Table 3, the linear regression analysis with the SASP index as the dependent variable and age, sex and the four factors as independent variables showed a significant effect of age (*P* < 0.001), sex (*P* < 0.001) and cardiovascular and cardiometabolic health (*P* < 0.001). Cardiovascular and cardiometabolic health was also the most significant independent variable when splitting our sample into males and females. This finding was supported when we repeated the regression analysis six times, excluding one of the covariates. As shown in Supplementary Table 6, the cardiovascular and cardiometabolic health factor contributed the most to the explained variance in the regression analysis. Including site as an additional covariate in the linear regression analysis did not significantly change the regression results (Supplementary Table 7).

### Influence of age of onset

We conducted additional exploratory analyses to test the influence of age of onset on the relationship between the SASP index and demographic and clinical variables. We split the sample into participants with early-onset depression (*n* = 356) or late-onset depression (*n* = 69). The information was missing for one participant. As displayed in Table 4, results were qualitatively similar for early-onset versus late-onset depression.

## Discussion

**Fig. 2 |:**
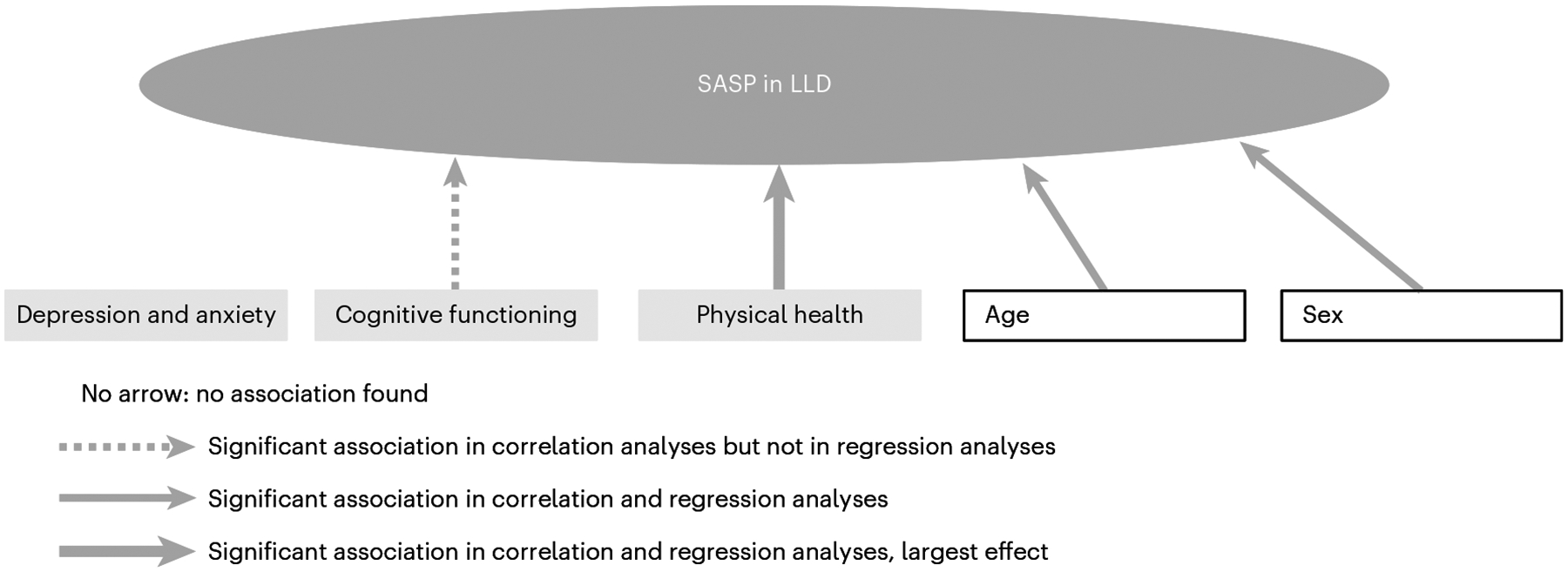
Summary of the results of the present study. Correlation analyses demonstrated significant associations between age/sex/cognitive functioning/physical health and the SASP. In addition, regression analyses showed a significant association between age/sex/physical health and the SASP, with physical health demonstrating the largest effects.

Previous work suggested a critical role of cellular senescence and an increased SASP index in LLD compared with non-depressed older adults^28,30,44^. The present study extended these findings by examining the variables related to the SASP index within individuals with LLD. Our results highlighted an association between an elevated SASP index and poor physical health. In addition, the SASP index was associated with age and sex, and cognitive functioning in females. In contrast, the SASP index was not associated with the severity of depression or anxiety (Fig. 2).

In individuals with LLD, the SASP index was most strongly associated with the cardiovascular and cardiometabolic health factor. Previous studies have demonstrated a robust bidirectional relationship between LLD and physical health^6^ and the mediating effect of medical conditions on treatment outcomes^45^. One previous study in young and middle-aged adults with major depressive disorder demonstrated that the SASP index was most strongly correlated to BMI^31^. In addition, pre-clinical studies have shown that fat cells express SASP proteins, which in turn cause a pro-oxidant, pro-inflammatory and pro-tumourigenic environment^46^. Obesity and diabetes mellitus have been linked to an increased expression of SASP proteins, and intervention studies have demonstrated that caloric restriction and exercise could delay cellular senescence and decrease SASP proteins^47^. Animal studies have shown that clearing senescent cells from obese mice restored neurogenesis, alleviating anxiety-related behaviour and chronic stress-induced cognitive decline^48^.

We observed a significant impact of age and biological sex on the SASP index. While cellular senescence occurs throughout life, senescent cells accumulate with age^44^ and contribute to tissue degradation. Therefore, our finding of a positive association between age and the SASP index is congruent with the description of biological senescence status as one of the hallmarks of ageing. While no studies have examined sex differences in SASP proteins, research on telomere length has reported sexual dimorphism^49^, and studies applying the allostatic load model to quantify physiological costs of stress found higher allostatic load in males than females^50^. In line with our findings, cardiovascular diseases develop later in females than males, and the association between LLD and increased mortality is particularly relevant for males^51^.

We found a significant but relatively weak correlation between the SASP index and cognitive functioning in females. Cognitive functioning—specifically executive functioning—has been identified as a predictor or moderator of treatment response in LLD^52^. In addition, one previous study on LLD reported a relationship between the SASP index and processing speed, executive functioning and general cognitive performance^30^. In another study, the SASP index was higher in individuals with comorbid LLD and mild cognitive impairments than in those with LLD without mild cognitive impairments^28^. These findings are expected, given the role of cellular senescence in brain functioning and neurological and neurodegenerative disorders^53^. While our sex-specific analyses must be interpreted with caution due to our larger number of female participants, they suggest that the association between the SASP index and cognition may be more robust in females.

Contrary to our hypothesis, the SASP index was not associated with the severity of depression or anxiety characteristics. When interpreting this finding, it is critical to note that it does not contradict our previous studies that have consistently demonstrated an increased SASP index in individuals with a major depressive disorder compared with non-depressed older adults^28,30,31^. However, it suggests that the SASP index is more closely associated with physical health and cognitive functioning than with the severity of depression and anxiety symptoms within individuals with LLD. The question of how depressive symptoms interact with the pathophysiology of major depression is fiercely discussed. For example, the severity and duration of depression are related to mortality^54^. Neuroimaging studies have reported a relationship between the age of onset and structural brain changes but not between symptom severity and structural abnormalities^55^. Some smaller studies examining telomere length have suggested a relationship between symptom severity and telomere length^56^, but other studies fail to report this association^57^ or only find it for younger adults. In one large longitudinal study of depressive symptoms, epigenetic changes and telomere length^58^, telomere length was shorter in individuals with depression. However, the authors did not find a relationship among changes in depressive symptoms, epigenetic markers or telomere length within an individual. They concluded that other features, such as inflammation, might drive the biological markers in depressed individuals. While this interpretation is also compelling for our findings, we acknowledge that differences in methods (for example, factor analyses versus single scores) might contribute to our findings. Additionally, previous studies suggested that other depression scales, such as the Geriatric Depression Scale or the Perceived Stress Scale, might be more sensitive to monitoring changes and treatment responses in LLD^59^. Therefore, longitudinal and more extensive studies are needed to explore the association between the SASP index and depression and anxiety characteristics within one individual.

Our findings highlight the interactive effect between LLD and physical burden. Previous research has demonstrated that depression frequently occurs in individuals with chronic illness and amplifies the disability and disablement associated with co-occurring physical illness and cognitive impairment^60^. In addition, depression undermines adherence to co-prescribed pharmacotherapy for medical diseases and reduces healthy lifestyle choices. Therefore, evidence-based treatment of depression may also reduce mortality risk secondary to physical illness, such as cancer^61^. On the other hand, co-occurring physical burden moderates the long-term response to antidepressant treatment and renders the individual’s response more brittle^45^. Last, the interaction of physical conditions and depression enhances the long-term risk for cognitive impairment and dementia. For these reasons, depression foreshortens life expectancy, while appropriate maintenance treatment may reduce mortality risk and increase life quality. Our findings suggest that future treatment efforts should include interventions and lifestyle modifications that target general health, such as weight loss and exercise programmes^62^, and optimized control of chronic medical conditions such as diabetes, hypertension and hypercholesterolaemia. Prescribed physical activity has been shown to prevent depression and has neuronal benefits^63^. In addition, geroscience-guided treatments that clear senescent cells or modify senescence and SASP-mediated pathways might provide promising avenues to improve LLD outcomes^64^. Future studies that examine the SASP longitudinally as a biomarker of ageing should examine if the SASP moderates long-term outcomes and if treatment of depression can modify the expression of SASP in a way that reduces the likelihood of physical and cognitive disabilities.

As highlighted, our study’s main limitation is that we did not include a healthy control group and did not collect longitudinal data. Therefore, we cannot test for causal relationships. Furthermore, future studies should build on our findings and use causal statistical models to examine the relationship among LLD, physical health and the SASP index. In addition, our analyses were limited by the nature of our data. For instance, the number of participants from under-represented groups was too small, and we could not assess the influence of race or ethnicity on the SASP index. However, previous studies have suggested associations between race/ethnicity and physical health or LLD^65^. Also, we did not have information on socioeconomic status except for the indirect measure of years of education. Similarly, we did not have information on childhood adversity or past traumas, which might mediate the relationship between neuropsychiatric symptoms and ageing. While we included a large selection of carefully pre-selected clinical measures, other variables might be relevant to capture the clinical heterogeneity of LLD. In particular, more detailed information on smoking and alcohol consumption, other physical comorbidities (for example, cerebrovascular disease) and general markers of quality of life, activities of daily living and physical activities should be included in future studies^66^.

In this study, we used the SASP index as a systemic measure of biological cellular senescence status. We have used the SASP index in several previous depression studies involving over 1,000 individuals and a longitudinal follow-up^28–31,67^. In addition, several other groups have used a subset of the SASP proteins and reported a relationship between these proteins and frailty, negative post-surgery outcomes or concussions^68,69^. However, senescence is a heterogeneous phenotype, probably dependent on the cell type^13,70^. While the cellular source of SASP proteins is unknown, some evidence suggests that the SASP index is relevant to brain health and that brain cells express SASP proteins^23–25^. In ageing and psychiatric disorders, the blood–brain barrier permeability is increased^71^, leading to an enhanced passage of proteins. Some studies suggest that plasma from old mice accelerates brain ageing in young mice^72^, and senolytic interventions targeting the periphery alleviate the effect of SASP proteins on the brain^71^. However, comparable studies in humans are missing. Future studies are needed to validate the SASP index and to compare its predictive accuracy with other indices of physical morbidity or biomarkers related to senescence and ageing. In addition, studies are needed to examine the relationship between the central and peripheral expression of SASP proteins.

## Conclusion

The present study aimed to understand the variables associated with biological senescence status and an increased SASP index in LLD. Physical health demonstrated the strongest association with the SASP index. In addition, age, sex and cognitive functioning were associated with the SASP index. The severity of depression and anxiety was unrelated to the SASP index. Thus, our findings highlight the importance of physical health for cellular senescence and ageing in LLD. Longitudinal studies are needed to examine the complex relationship among LLD, physical health and ageing.

## Methods

### Study sample

We included 426 participants, 60 years old or older, with a diagnosis of current non-psychotic major depressive disorder and a score ≥15 on the MADRS^36^. The present study presents a secondary data analysis of the baseline data of a completed study ((IRL-GREY), ClinicalTrials.gov Identifier: NCT00892047). The present study was a secondary data analysis of the parent IRL-GREY study^35^. Therefore, we did not carry out power calculations for this secondary data analysis but included all participants with plasma samples from the parent study (426 out of 468).

Participant’s assessment took place from 20 July 2009 to 30 December 2013 in research offices at three sites: the University of Pittsburgh, Pittsburgh, PA; Washington University at St Louis, St Louis, MO; and the Centre for Addiction and Mental Health, Toronto, ON, Canada. The study protocol can be found at https://www.clinicaltrials.gov/ct2/show/NCT02960763?term=Eric+Lenze&draw=2&rank=3. Ethics approval was obtained from the institutional review boards at the Washington University at St Louis, the University of Pittsburgh Medical Center and the Centre for Addiction and Mental Health. Additional approval was obtained from the institutional review board at the University of Connecticut Health Center for biomarker analyses. All participants provided written informed consent and were compensated for participating in the IRL-GREY study.

The diagnosis of major depressive disorder and the presence of a major depressive episode was based on the Diagnostic and Statistical Manual of Mental Disorders, 4th Edition, Text Revision (DSM-IV-TR) diagnostic criteria^73^ and confirmed with the Structured Clinical Interview for DSM-IV-TR Axis I Disorders (SCID-IV-TR)^74^. Exclusion criteria were lifetime diagnosis of bipolar disorder, schizophrenia, schizoaffective disorder, other psychotic disorders, current psychotic symptoms, clinical history of dementia, alcohol or substance abuse in the past 6 months, imminent suicide risk, unstable physical illness or contraindication to venlafaxine XR. Ethics approval was obtained from the institutional review boards at the three participating sites (no. 201712078), and all participants provided written informed consent. In this paper, we included only data related to the baseline assessment before the initiation of venlafaxine XR.

### Clinical assessments

We obtained age, self-reported sex and self-reported race for all individuals.

#### Depression and anxiety characteristics.

Characteristics of LLD, including the length of the current depressive episode, age of onset of the first depressive episode, recurrence, previous suicide attempts and comorbid anxiety disorders, were obtained during the SCID-IV-TR interview. The severity of depressive symptoms was evaluated by the total score on the MADRS^36^. Anxiety symptoms and severity were assessed by the ASI^37^. We evaluated suicidality with the SSI^38^. We also included the Medical Outcomes Survey—mental^39^ to assess mental well-being and quality of life.

#### Cognitive functioning.

We recorded self-reported years of education for all individuals. Global cognitive performance was evaluated with the MMSE^40^ and the RBANS (global cognitive performance domain)^41^. Given that the RBANS does not include executive functioning, we also included the DKEFS Trail Making Test (condition 4 versus condition 5) and the DKEFS Color Word Inference (final condition 3) (executive function domain). We utilized the mean of these two variables for further analyses^42^.

#### Physical health status.

We obtained anthropometric data and calculated BMI (kg m^2^). Blood pressure was measured while sitting after resting for at least 5 min, with systolic and diastolic blood pressure values being the mean of two measurements taken 5 min apart. We determined fasting glucose levels for all individuals. We assessed the burden of physical illness with the CIRS-G^43^ and the Medical Outcomes Survey—physical^39^.

#### Medication.

We classified self-reported medication following the first level category of the Anatomical Therapeutic Chemical Classification. We provide a more detailed overview of psychoactive drugs, including anticonvulsants, antidepressants, antipsychotics, anxiolytics/sedatives/hypnotics, opiates and stimulants.

### Laboratory analyses of the SASP proteins

Blood was collected by venipuncture with ethylenediaminetetraacetic acid tubes after overnight fasting and processed within 3 h of collection using standard procedures. Plasma was obtained from the blood by centrifugation at 3,000*g* for 10 min at 4 °C, aliquoted and stored in a −80 °C freezer until the laboratory analyses.

The SASP proteins were analysed by a customized multiplex assay (R&D Systems) using the Luminex platform LX 100/200 (Luminex). All laboratory personnel responsible for the analyses were blinded to demographic and clinical data. We performed all experiments according to the manufacturer’s instructions. All samples were analysed using the same assay batch on the same day to reduce variability, and the coefficient of variation was <10% for all analyses.

Given the heterogeneity of senescence and its lack of dependence on specific cells, there is no established SASP panel. However, previous research suggests that senescence markers are related to inflammation, extracellular matrix, signalling and growth factors^75^. On the basis of this work, we have developed a SASP index comprising 22 proteins and validated it in several studies^30^, including 527 individuals with depression and 638 healthy individuals^31^, and another study of 371 older individuals to examine the influence of depression and mild cognitive impairments on the SASP index^28^. We have also reported that the SASP index in depression relates to brain structural abnormalities^67^. Finally, in a longitudinal study of a subsample of the participants included in the present analysis, we have shown that a higher SASP index at baseline independently predicted a lower remission rate to venlafaxine treatment^29^.

As in our previous studies^28,30^, the 22 proteins included in the SASP index used in the present study are IGFBP-6, IGFBP-2, MIP-1β, IL-1β, GMC-SF, PLGF, Angiogenin, MIF-1, MIP-1α, Gro-α, IL-6, MCP-4, Gp130, ICAM-1, MCP-1, IL-8, MIP-3 α, Osteoprotegerin, TIMP-1, uPAR, TNFRI and TNFRII. The raw values were log transformed and standardized to *z* scores, and the SASP index for each participant was calculated based on the following regression formula:

$$\text{SASP index}=\beta_{1}x_{1}+\ldots +\beta_{22}x_{22}$$

where *β* is the individual weight and *x* is the standardized *z* score of each protein included in the SASP index. The weights for each biomarker were derived from our previous study in an independent sample of older adults with and without a history of major depressive disorder (Supplementary Table 8) (ref.^28^).

### Statistical analyses

Statistical analyses were performed using IBM SPSS Statistics 27 and GraphPad Prism 9. We only included participants for whom we could calculate the SASP index. We had complete demographic data for all participants. However, some participants were missing some clinical variables. Therefore, we used linear imputations for missing data procedures provided in IBM SPSS Statistics 27 and included all participants in our primary analyses. The last value before and the first after the missing value are used for the interpolation. If the first or last case in the series has a missing value, the missing value is not replaced.

Our analytic strategy consisted of four steps:

1. Clinical variables: following previous studies^32^, we conducted factor analyses to group clinical variables into different domains. It has been suggested that integrating multiple variables using factor analyses is more reliable when estimating disease trajectories^33^ and determining treatment outcomes^34^ than using single variables. Here, we conducted separate factor analyses, including variables related to (1) depression and anxiety characteristics, (2) cognitive functioning and (3) physical health. The assumptions for conducting factor analyses were tested using Bartlett’s sphericity tests. We utilized the Anderson–Rubin method to extract factors and Varimax rotation to ensure the orthogonality of the estimated factors. Only factors with an eigenvalue above one were extracted.
2. Relationship between the SASP index and demographic variables: we conducted a regression analysis with the SASP index as the dependent variable and age and sex as independent variables. We tested for normality using a normal probability plot of regression standardized residual. We calculated the variance inflation factor to detect multicollinearity between the independent variables. A *P* value less than 0.025 was considered significant.
3. Relationship between the SASP index and clinical variables: first, we used correlation analyses to explore the association between the SASP index and the factors determined in step one. In addition, we computed correlation analyses separately for males and females to test for a sex-specific association between the SASP index and the factors. We computed Pearson and Spearman correlations because not all variables were normally distributed. *P* values were Bonferroni corrected for the number of correlations computed, and a *P* value less than 0.0013 was considered significant. Next, we conducted a linear regression analysis with the SASP index as the dependent variable, and age, sex and all factors as independent variables. We tested for normality using a normal P-P plot of regression standardized residual. We calculated the variance inflation factor to detect multicollinearity between the independent variables. To determine if an independent variable had a significant effect on the SASP, a Bonferroni-corrected *P* value less than 0.0083 was considered significant. We repeated the regression analysis for males and females separately. In sensitivity analyses, we first repeated the regression analysis six times, each time excluding one of the covariates to estimate the contribution of each independent variable separately. Finally, we repeated the regression analyses, including the site (Pittsburgh, Toronto or Washington) as an additional covariate.
4. Influence of age of onset on the relationship between the SASP index and clinical variables: We conducted additional exploratory analyses to assess whether the age of onset influences the relationships above. We repeated the regression analysis for participants with early-onset (before 65 years) versus late-onset (65 years and older) depression separately.

Please note that all reported tests were two tailed.

## Supplementary Material

supplementary material

## Figures and Tables

**Table 1 |:** Demographic and clinical variables (categorical variables)

Categorical variables		
	*N*	Distribution
**Demographic variables**		
Sex	426	Female: 273 (64%)
		Male: 153 (36%)
Site	426	Pittsburgh: 175 (41%)
		Toronto: 119 (28%)
		Washington: 132 (31%)
Race	426	Asian Pacific: 7 (2%)
		Black: 40 (9%)
		Native American: 1 (<1%)
		White: 378 (89%)
Alcohol consumption^a^	426	Yes: 212 (50%)
		No: 214 (50%)
**Variables related to depression and anxiety characteristics**		
Depression type	426	Recurrent: 307 (72%)
		Single episode: 119 (28%)
Previous suicide attempts	424	Yes: 52 (12%)
		No: 372 (87%)
SCID anxiety diagnosis	426	Yes: 178 (42%)
		No: 248 (58%)

a Alcohol consumption: the estimation is based on the number of individuals who reported consuming alcohol during the clinical trial following baseline data collection.

**Table 2 |:** Demographic and clinical variables (continuous variables)

Continuous variables					
	*N*	Mean	Standard deviation	Minimum	Maximum
**Demographic variables**					
Age	426	68.94	7.10	60.05	93.07
**Variables related to depression and anxiety characteristics**					
Age of onset (years)	425	42.03	21.30	4	91
Duration of illness (months)	424	292.25	617.58	2	3744
MADRS^36^	426	26.58	5.72	13	43
ASI^37^	421	25.64	12.77	1	59
SSI	425	2.31	4.47	0	24
Medical Outcomes Survey—mental^39^	419	27.36	8.84	4.16	57.34
**Variables related to cognitive functioning**					
Years of education	426	14.45	2.87	6	20
MMSE^40^	369	28.66	1.54	21	30
RBANS^41^	413	95.40	15.78	47	148
DKEFS^42^	418	9.24	2.85	1	16
**Variables related to physical health**					
BMI	422	29.68	6.76	17.29	57.37
Sitting systolic blood pressure	419	132.14	19.12	90	199
Sitting diastolic blood pressure	419	76.27	11.58	47	115
Glucose	425	110.30	40.75	9.40	341.00
CIRS-G^43^	425	9.94	4.47	0	26
Medical Outcomes Survey—physical^39^	419	42.36	11.63	15.70	71.74

**Table 3 |:** Two-tailed regression analyses

	Standardized *β*	*T*	*P*	Collinearity statistics: variance inflation factor^a^
**All participants**				
*F*(6, 419) = 24.56, *P* < 0.001, adjusted *R*^2^ = 0.25 and power >0.99				
Age	0.18	4.17	<0.001	1.10
Sex	−0.18	−4.27	<0.001	1.03
Depression and anxiety severity	−0.038	−0.89	0.38	1.05
Cognitive functioning	−0.086	−1.96	0.051	1.08
Cardiovascular and cardiometabolic health	0.39	9.25	<0.001	1.02
Blood pressure	0.027	0.63	0.53	1.02
**Male participants**				
*F*(5,147) = 12.98, *P* < 0.001, adjusted *R*^2^ = 0.28 and power >0.99				
Age	0.13	1.75	0.082	1.12
Depression and anxiety severity	−0.091	−1.29	0.20	1.06
Cognitive functioning	−0.020	−0.27	0.79	1.12
Cardiovascular and cardiometabolic health	0.50	6.76	<0.001	1.14
Blood pressure	−0.018	−0.25	0.80	1.04
**Female participants**				
*F*(5, 267) = 14.32, *P* < 0.001, adjusted *R*^2^ = 0.20 and power >0.99				
Age	0.22	3.79	<0.001	1.10
Depression and anxiety severity	−0.013	−0.24	0.81	1.04
Cognitive functioning	−0.12	−2.06	0.041	1.08
Cardiovascular and cardiometabolic health	0.35	6.38	<0.001	1.02
Blood pressure	0.074	1.33	0.18	1.03

We conducted two-tailed regression analyses, with the SASP index as the dependent variable and age, sex and the factors as independent variables. *P* values less than 0.0083 (Bonferroni correction for six covariates) are considered significant and bolded.

a The variance inflation factor is used to detect multicollinearity. The value starts at 1 and has no upper limit. A general rule of thumb is that values between 1 and 5 indicate some correlations that are not problematic enough to require attention (for correlations between factors, see Supplementary Table 5).

**Table 4 |:** Regression analyses, stratified by early-onset versus late-onset depression

	Standardized *β*	*T*	*P*	Collinearity statistics: variance inflation factor^a^
**Early-onset depression (age of onset before 65 years)**				
*F*(6, 349) = 19.02, *P* < 0.001, adjusted *R*^2^ = 0.23 and power >0.99				
Age	0.13	2.65	0.008	1.06
Sex	−0.20	−4.24	<0.001	1.02
Depression and anxiety severity	−0.030	−0.65	0.52	1.03
Cognitive functioning	−0.082	−1.73	0.084	1.05
Cardiovascular and cardiometabolic health	0.40	8.55	<0.001	1.02
Blood pressure	0.030	0.64	0.52	1.02
**Late-onset depression (age of onset at or after 65 years)**				
*F*(6, 62) = 3.53, *P* = 0.005, adjusted *R*^2^ = 0.18 and power 0.82				
Age	0.25	2.11	0.039	1.14
Sex	−0.14	−1.15	0.25	1.20
Depression and anxiety severity	−0.089	−0.77	0.45	1.11
Cognitive functioning	−0.095	−0.82	0.41	1.11
Cardiovascular and cardiometabolic health	0.39	3.40	<0.001	1.10
Blood pressure	−0.012	−0.10	0.92	1.11

We conducted additional exploratory two-tailed regression analyses to assess whether the age of onset influences the relationships above. *P* values less than 0.0083 (Bonferroni correction for six covariates) are considered significant and bolded.

a The variance inflation factor is used to detect multicollinearity. The value starts at 1 and has no upper limit. A general rule of thumb is that values between 1 and 5 indicate some correlations that are not problematic enough to require attention.

## Data Availability

The current data related to the biomarkers analyses from the IRL-GREY study can be shared upon request from investigators and after approval from all investigators associated with the IRL-GREY Biomarkers Study. Please contact B.S.D. at diniz@uchc.edu.
