# Supplementary material for: Major depression, physical health and molecular senescence markers abnormalities
Source: Nat Ment Health. Author manuscript; Available in PMC 2024 Oct 31. (PMC11527398; doi:10.1038/s44220-023-00033-z)
Supplement: supplementary material [file NIHMS1989206-supplement-supplementary_material.pdf]

nature mental health

Article https://doi.org/10.1038/s44220-023-00033-z

Major depression, physical health and molecular senescence markers abnormalities

In the format provided by the authors and unedited

Supplementary Material

Supplementary Tables

Supplementary Table 1: CONSORT 2010 checklist of information to include when reporting a randomised trial

Section/Topic	Item No	Checklist item	Reported on page No
**Title and abstract**			
	1a	Identification as a randomised trial in the title	NA
	1b	Structured summary of trial design, methods, results, and conclusions (for specific guidance see CONSORT for abstracts)	NA
**Introduction**			
Background and objectives	2a	Scientific background and explanation of rationale	4, 5
	2b	Specific objectives or hypotheses	5, 6
**Methods**			
Trial design	3a	Description of trial design (such as parallel, factorial) including allocation ratio	NA
	3b	Important changes to methods after trial commencement (such as eligibility criteria), with reasons	NA
Participants	4a	Eligibility criteria for participants	15
	4b	Settings and locations where the data were collected	15
Interventions	5	The interventions for each group with sufficient details to allow replication, including how and when they were actually administered	NA
Outcomes	6a	Completely defined pre-specified primary and secondary outcome measures, including how and when they were assessed	NA
	6b	Any changes to trial outcomes after the trial commenced, with reasons	NA
Sample size	7a	How sample size was determined	15
	7b	When applicable, explanation of any interim analyses and stopping guidelines	NA
Randomisation:			
Sequence generation	8a	Method used to generate the random allocation sequence	NA
	8b	Type of randomisation; details of any restriction (such as blocking and block size)	NA

Allocation concealment mechanism	9	Mechanism used to implement the random allocation sequence (such as sequentially numbered containers), describing any steps taken to conceal the sequence until interventions were assigned	NA
Implementation	10	Who generated the random allocation sequence, who enrolled participants, and who assigned participants to interventions	NA
Blinding	11a	If done, who was blinded after assignment to interventions (for example, participants, care providers, those assessing outcomes) and how	NA
	11b	If relevant, description of the similarity of interventions	NA
Statistical methods	12a	Statistical methods used to compare groups for primary and secondary outcomes	19, 20
	12b	Methods for additional analyses, such as subgroup analyses and adjusted analyses	19, 20
**Results**			
Participant flow (a diagram is strongly recommended)	13a	For each group, the numbers of participants who were randomly assigned, received intended treatment, and were analysed for the primary outcome	NA
	13b	For each group, losses and exclusions after randomisation, together with reasons	NA
Recruitment	14a	Dates defining the periods of recruitment and follow-up	15
	14b	Why the trial ended or was stopped	NA
Baseline data	15	A table showing baseline demographic and clinical characteristics for each group	25, 26
Numbers analysed	16	For each group, number of participants (denominator) included in each analysis and whether the analysis was by original assigned groups	25, 26
Outcomes and estimation	17a	For each primary and secondary outcome, results for each group, and the estimated effect size and its precision (such as 95% confidence interval)	NA
	17b	For binary outcomes, presentation of both absolute and relative effect sizes is recommended	NA
Ancillary analyses	18	Results of any other analyses performed, including subgroup analyses and adjusted analyses, distinguishing pre-specified from exploratory	7, 8
Harms	19	All important harms or unintended effects in each group (for specific guidance see CONSORT for harms)	NA
**Discussion**			
Limitations	20	Trial limitations, addressing sources of potential bias, imprecision, and, if relevant, multiplicity of analyses	13
Generalisability	21	Generalisability (external validity, applicability) of the trial findings	9-11
Interpretation	22	Interpretation consistent with results, balancing benefits and harms, and considering other relevant evidence	8-14
**Other information**			

Registration	23	Registration number and name of trial registry	15
Protocol	24	Where the full trial protocol can be accessed, if available	15
Funding	25	Sources of funding and other support (such as supply of drugs), role of funders	22, 23

Supplementary Table 2: Medication

Anatomical Therapeutic Chemical (ATC) Classification, 1st level category	Absolute Number	Percentage of individuals
A: Alimentary tract and metabolism	314	74%
Drugs used in diabetes	78	18%
Vitamins, Minerals, and Supplements	217	51%
B: Blood and blood-forming organs	54	13%
C: Cardiovascular system	280	66%
D: Dermatologicals	20	5%
G: Genito urinary system and sex hormones	48	11%
H: Systemic hormonal preparation, excluding sex hormones and insulins	92	22%
J: Anti-infective for systemic use	21	5%
L: Antineoplastic and immunomodulating agents	11	3%
M: Musculoskeletal system	258	61%
N: Nervous system	285	67%
Anticonvulsant agent	31	7%
Antidepressant	170	40%
Antipsychotic agent	4	1%
Anxiolytics, Sedatives, Hypnotics	160	38%
Opiates	70	16%
Stimulants	1	<1%
P: Antiparasitic products, insecticides, and repellents	3	<1%
R: Respiratory System	133	31%
S: Sensory organs	4	1%

Medication use is based on self-reported medication at baseline.

Supplementary Table 3: Factor analyses

**Variables related to depression and anxiety characteristics**		
Bartlett's sphericity test: χ2(6) = 179.62, p < .001		
	**Depression and anxiety severity**	
Montgomery-Asberg Depression Rating Scale [15]	**0.81**	
Anxiety Sensitivity Index [19]	**0.59**	
Scale of Suicidal Ideation	**0.47**	
Medical Outcomes Survey- Mental [21]	**-0.76**	
Eigenvalue	1.79	
Variance accounted	44.79%	
**Variables related to cognitive functioning**		
Bartlett's sphericity test: χ2(6) = 367.18, p < .001		
	**Cognitive functioning**	
Years of education	**.64**	
Mini-Mental Status Examination [22]	**.71**	
Repeatable Battery for the Assessment of Neuropsychological Status [23]	**.84**	
Delis-Kaplan Executive Function System [24]	**.78**	
Eigenvalue	2.23	
Variance accounted	55.64%	
**Variables related to physical health**		
Bartlett's sphericity test: χ2(15) = 360.40, p < .001		
	**Cardiovascular and cardiometabolic health**	**Blood pressure**
Body mass index	**.61**	.27
Sitting systolic blood pressure	.17	**.86**
Sitting diastolic blood pressure	-.14	**.89**
Glucose	**.47**	-.011
Cumulative Illness Rating Scale – Geriatrics [25]	**.77**	-.036
Medical Outcomes Survey- Physical [21]	**-.74**	.048
Eigenvalue	1.84	1.54
Variance accounted	30.70%	25.69%

We conducted separate factor analyses, including variables related to 1) depression and anxiety characteristics, 2) cognitive functioning, and 3) physical health. The assumptions for conducting factor analyses were tested using Bartlett's sphericity tests. We utilized the Anderson-Rubin method to extract factors and Varimax rotation to ensure the orthogonality of the estimated factors. Only factors with an eigenvalue above one were extracted. Factor loadings higher than 0.30 are highlighted **in bold.**

Supplementary Table 4: Correlations between SASP index and factors

	Pearson	Spearman
**All participants** (n = 426)		
Depression and anxiety severity	R = -0.091, p = .059, Power = 0.27	Rho = -0.078, p = 0.11, Power = 0.19
Cognitive functioning	**R = - 0.18, p < .001,** Power = 0.90	**Rho = -0.20, p < .001,** Power = 0.95
Cardiovascular and cardiometabolic health	**R = 0.42, p < .001,** Power > 0.99	**Rho = 0.41, p < .001,** Power > 0.99
Blood pressure	R =0.027, p = 0.58, Power > 0.99	Rho = 0.0080, p = 0.87, Power = 0.014
**Male participants (**n = 153)		
Depression and anxiety severity	R = -0.11, p = 0.17, Power = 0.13	Rho = -0.055, p = 0.50, Power = 0.036
Cognitive functioning	R = -0.19, p = 0.021, Power = 0.45	Rho = -0.17, p = 0.033, Power = 0.35
Cardiovascular and cardiometabolic health	**R = 0.53, p < .001,** Power > 0.99	**Rho = 0.51, p < .001,** Power > 0.99
Blood pressure	R = -0.12, p = 0.16, Power = 0.16	Rho = -0.12, p = 0.13, Power = 0.16
**Female participants** (n = 273)		
Depression and anxiety severity	R = -0.059, p = 0.33, Power = 0.066	Rho = -0.064, p = 0.29, Power = 0.076
Cognitive functioning	**R = -0.17, p = 0.0050,** Power = 0.63	**Rho = - 0.20, p < .001,** Power = 0.79
Cardiovascular and cardiometabolic health	**R = 0.37, p < .001,** Power > 0.99	**Rho = 0.38, p <.001**, Power > 0.99
Blood pressure	R = 0.082, p = 0.18, Power = 0.13	Rho = 0.060, p = 0.32, Power = 0.067

We used two-tailed Pearson and Spearman correlation analyses to explore the association between the SASP index and the factors determined. P-values smaller than 0.013 (Bonferroni correction for four tests) are considered significant and **bolded.**

Power calculations are based on Fisher's z-transformation and normal approximation with bias adjustment for Pearson correlations and Fisher's z-transformation, normal approximation, and variance estimation (Bonett and Wright) for Spearman correlations.

Abbreviations: senescence-associated secretory phenotype (SASP)

Supplementary Table 5: Correlations between factors

	Depression and anxiety severity	Cognitive functioning	Cardiovascular and cardiometabolic health	Blood pressure
Depression and anxiety severity				
Cognitive functioning	R = -0.043, p = 0.38, Power = 0.055			
Cardiovascular and cardiometabolic health	R = -0.028, p. = 0.57, Power = 0.029	R = -0.12, p = 0.010, Power = 0.50		
Blood pressure	R = 0.001, p = 0.98, Power = 0.013	R = 0.059, p = 0.22, Power = 0.10	R < 0.001, p > .99, Power = 0.013	

We used two-tailed Pearson correlation analyses to explore the association between the factors. P-values smaller than 0.0083 (Bonferroni correction for six tests) are considered significant and **bolded.**

Power calculations are based on Fisher's z-transformation and normal approximation with bias adjustment.

Supplementary Table 6: Explained variance in regression analyses, dependent variable SASP

Included variables	Statistics
F(6, 419) = 24.56, p <.001, adjusted R^2 = 0.25, Power > 0.99	
• Age	• **Standardized Beta = 0.18, T = 4.17, p < .001**
• Sex	• **Standardized Beta = -0.18, T = -4.27, p < .001**
• Depression and anxiety severity	• Standardized Beta = -0.038, T = -0.89, p = 0.38
• Cognitive functioning	• Standardized Beta = -0.086, T = -1.96, p = 0.051
• Cardiovascular and cardiometabolic health	• **Standardized Beta = 0.39, T = 9.25, p < .001**
• Blood pressure	• Standardized Beta = 0.027, T = 0.63, p = 0.53
F(5, 420) = 25.02, p <.001, adjusted R^2 = 0.22, Power > 0.99	
• Sex	• **Standardized Beta = -0.17, T = -3.92, p < .001**
• Depression and anxiety severity	• Standardized Beta = -0.073, T = -1.69, p = 0.092
• Cognitive functioning	• Standardized Beta = -0.13, T = -2.93, p = 0.004
• Cardiovascular and cardiometabolic health	• **Standardized Beta = 0.40, T = 9.32, p < .001**
• Blood pressure	• Standardized Beta = 0.018, T = 0.42, p = 0.68
F(5, 420) = 24.80, p <.001, adjusted R^2 = 0.22, Power > 0.99	
• Age	• **Standardized Beta = 0.17, T = 3.81, p < .001**
• Depression and anxiety severity	• Standardized Beta = -0.055, T = -1.27, p = 0.21
• Cognitive functioning	• Standardized Beta = -0.10, T = -2.32, p = 0.021
• Cardiovascular and cardiometabolic health	• **Standardized Beta = 0.39, T = 8.97, p < .001**
• Blood pressure	• Standardized Beta = 0.045, T = 1.04, p = 0.30
F(5, 420) = 29.33, p <.001, adjusted R^2 = 0.25, Power > 0.99	
• Age	• **Standardized Beta = 0.19, T = 4.43, p < .001**
• Sex	• **Standardized Beta = - 0.19, T = -4.38, p < .001**
• Cognitive functioning	• Standardized Beta = -0.082, T = -1.88, p = 0.060
• Cardiovascular and cardiometabolic health	• **Standardized Beta = 0.39, T = 9.27, p < .001**
• Blood pressure	• Standardized Beta = 0.027, T = 0.63, p = 0.53
F(5, 420) = 28.52, p <.001, adjusted R^2 = 0.25, Power > 0.99	
• Age	• **Standardized Beta = 0.20, T = 4.72, p < .001**
• Sex	• **Standardized Beta = - 0.19, T = -4.46, p < .001**
• Depression and anxiety	• Standardized Beta = -0.030, T = -0.70, p = 0.48
• Cardiovascular and cardiometabolic health	• **Standardized Beta = 0.40, T = 9.49, p < .001**
• Blood pressure	• Standardized Beta = 0.022, T = 0.52, p = 0.60
F(5, 420) = 10.31, p <.001, adjusted R^2 = 0.10, Power > 0.99	
• Age	• **Standardized Beta = 0.21, T = 4.29, p < .001**
• Sex	• **Standardized Beta = - 0.17, T = -3.71, p < .001**

• Depression and anxiety	• Standardized Beta = -0.048, T = -1.01, p = 0.31
• Cognitive functioning	• **Standardized Beta = -0.13, T = -2.74, p = 0.006**
• Blood pressure	• Standardized Beta = 0.032, T = 0.68, p = 0.49
F(5, 420) = 29.44, p <.001, adjusted R^2 = 0.25, Power > 0.99	
• Age	• **Standardized Beta = 0.18, T = 4.15, p < .001**
• Sex	• **Standardized Beta = - 0.19, T = -4.36, p < .001**
• Depression and anxiety	• Standardized Beta = -0.038, T = -0.89, p = 0.38
• Cognitive functioning	• Standardized Beta = -0.084, T = -1.93, p = 0.054
• Cardiovascular and cardiometabolic health	• **Standardized Beta = 0.39, T = 9.26, p < .001**

We conducted a two-tailed linear regression analysis with the SASP index as the dependent variable and age, sex, and all factors as independent variables. To determine if an independent variable had a significant effect on the SASP, a Bonferroni-corrected p-value smaller than 0.0083 was considered significant (Bonferroni correction for six covariates). P-values smaller than 0.0083 are **bolded.**

Abbreviations: senescence-associated secretory phenotype (SASP)

Supplementary Table 7: Regression analyses with the site as an additional covariate, dependent variable SASP

	Standardized Beta	T	P	Collinearity statistics: variance inflation factor *
**All participants**				
F(7, 418) = 21.86, p <.001, adjusted R^2 = 0.26, Power > 0.99				
Age	0.20	4.42	**< .001**	1.12
Sex	-0.18	-4.23	**<.001**	1.03
Depression and anxiety severity	-0.043	-1.00	0.32	1.05
Cognitive functioning	-0.076	-1.72	0.085	1.10
Cardiovascular and cardiometabolic health	0.41	9.52	**< .001**	1.07
Blood pressure	0.040	0.94	0.35	1.04
Site	0.093	2.11	0.035	1.10

We conducted a two-tailed linear regression analysis with the SASP index as the dependent variable and age, sex, all factors, and site as independent variables. To determine if an independent variable had a significant effect on the SASP, a Bonferroni-corrected p-value smaller than 0.0071 was considered significant (Bonferroni correction for seven covariates). P-values smaller than 0.0071 are **bolded.**

* The variance inflation factor is used to detect multicollinearity. The value starts at one and has no upper limit. A general rule of thumb is that values between 1 and 5 indicate some correlations that are not problematic enough to require attention.

Abbreviation: senescence-associated secretory phenotype (SASP)

Supplementary Table 8: Weights to construct the SASP index

Proteins	Weights
Insulin growth factor binding protein 6 (IGFBP-6)	-0.185
Insulin growth factor binding protein 6 (IGFBP-2)	-0.104
Macrophage Inflammatory Protein 1β (MIP-1β, CCL4)	0.556
Interleukin 1 beta (IL-1β)	0.536
Granulocyte-macrophage colony-stimulating factor (GMC-SF, CSF2)	0.466
Placental growth factor (PLGF)	0.211
Angiogenin	-0.364
Macrophage Inhibitory Factor-1 (MIF-1)	0.304
Macrophage Inflammatory Protein 1β α (MIP-1 α, CCL3)	0.572
Chemokine (C-X-C motif) ligand 1 (CXCL1, GRO-α)	0.169
Interleukin 6 (IL-6)	0.364
Chemokine (C-C motif) ligand 13 (CCL13, MCP-4)	0.347
Glycoprotein 130 (Gp130)	0.403
Intercellular Adhesion Molecule 1 (ICAM-1)	0.416
chemokine (C-C motif) ligand 2 (CCL2, MCP-1)	0.35
Interleukin 8 (IL-8)	0.47
Chemokine (C-C motif) ligand 20 (CCL20, MIP-3α)	0.481
Osteoprotegerin	0.565
TIMP metallopeptidase inhibitor 1 (TIMP-1)	-0.219
Urokinase-type plasminogen activator receptor (uPAR)	0.583
Tumor necrosis factor receptor 1 (TNFR1)	0.719
Tumor necrosis factor receptor 2 (TNFR2)	0.612

These SASP proteins were selected based on previous preclinical studies focused on the changes in the secretome pattern of senescent cells [26] and our previous publications [8,9]. Weights are derived from a previous publication from our group using an independent sample of older adults with and without a history of major depressive disorder [9].
Abbreviation: senescence-associated secretory phenotype (SASP)

Supplementary Figures

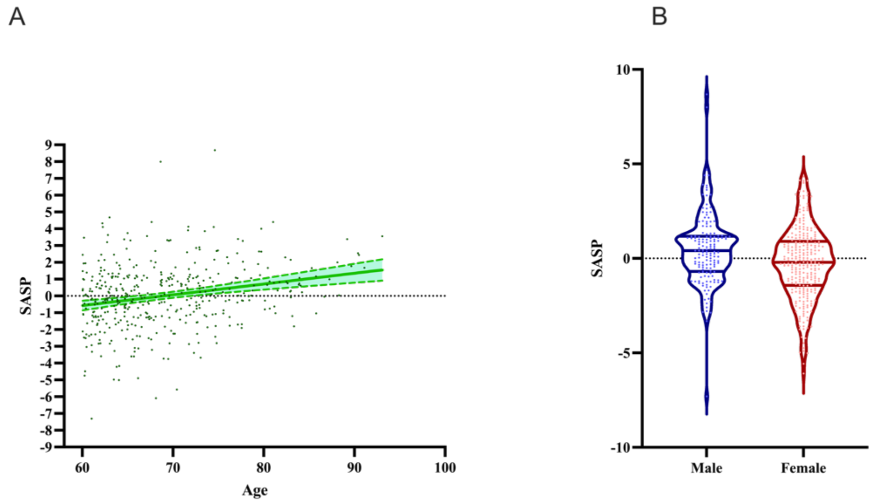

Supplementary Figure 1 displays the relationship between the senescence-associated secretory phenotype (SASP) index and demographic and clinical variables.

A) Older individuals demonstrated a higher SASP index than younger individuals. The bold line presents the best linear regression fit. The dotted line presents the 95% confidence interval. B) In addition, males displayed a higher SASP index than females. Violin plots were chosen to display a) the median, b) the interquartile range, and c) single data points.
